# Environmental Quality, Extreme Heat, and Healthcare Expenditures

**DOI:** 10.3390/ijerph21101322

**Published:** 2024-10-05

**Authors:** Douglas A. Becker

**Affiliations:** Conservation International, Arlington, VA 22202, USA; dbecker@conservation.org

**Keywords:** environmental quality, EQI, air temperature, healthcare spending

## Abstract

Although the effects of the environment on human health are well-established, the literature on the relationship between the quality of the environment and expenditures on healthcare is relatively sparse and disjointed. In this study, the Environmental Quality Index developed by the Environmental Protection Agency and heatwave days were compared against per capita Medicare spending at the county level. A general additive model with a Markov Random Field smoothing term was used for the analysis to ensure that spatial dependence did not undermine model results. The Environmental Quality Index was found to hold a statistically significant (*p* < 0.05), multifaceted nonlinear association with spending, as was the average seasonal maximum heat index. The same was not true of heatwave days, however. In a secondary analysis on the individual domains of the index, the social and built environment components were significantly related to spending, but the air, water, and land domains were not. These results provide initial support for the simultaneous benefits of healthcare financing systems to mitigate some dimensions of poor environmental quality and consistently high air temperatures.

## 1. Introduction

How the environment affects human health has been studied exhaustively and is now well understood. Good environmental quality facilitates good health, and poor environmental quality can cause substantial excess disease, disability, and death [1,2,3]. The environment can affect human health in myriad, drastic, and expensive ways [4]. Less research has been conducted on how health effects wrought by the environment extend to healthcare spending. A better understanding of the relationship between the environment as a whole and spending can allow us to assess the economic impacts of adverse environmental conditions, perform cost–benefit analyses of environmental management strategies and/or regulations, and provide substantive improvements to the burdensome levels of healthcare spending in the US more accurately. Moreover, human beings live amid the entirety of the environment and not just parts of it, so an analysis that considers the total environment more accurately reflects reality.

The existing literature on environmental quality and healthcare spending is dominated by studies on certain facets of the environment—such as air or water quality—or specific individual pollutants. Air quality and spending is probably the most researched relationship. Research on this link has shown savings in healthcare cost due to a reduction in aggregate air pollution across several respiratory and cardiovascular health outcomes [5]. Specific air pollutants, such particulate matter (both fine and coarse), ozone, nitrogen oxides, sulfur dioxide, and carbon monoxide have been linked to higher healthcare costs [6]. Environmental tobacco smoke, or second-hand smoke, also leads to more healthcare spending [7,8].

Various dimensions of water quality have also been linked to increased healthcare spending, most commonly disease outbreaks attributable to waterborne pathogens. Waterborne disease outbreaks in Finland [9] and Wisconsin [10] have been associated with substantial excessive healthcare costs. It has been estimated that surface water contamination in the United States results in approximately USD 3 billion in costs every year, most of which is attributable to the costs of caring for the victims [11]. Increased healthcare expenditures are associated with the number of per capita violations of the Drinking Water Act in the US [12].

Air temperature also unsurprisingly impacts healthcare spending. Both hypothermia and hyperthermia drive healthcare expenditures up by dozens or even hundreds of thousands of dollars per hospital visit [13]. Extreme heat events, also known as heatwaves, result in significantly higher healthcare costs, especially among the elderly [14]. Multiple studies have used carbon dioxide emission levels to represent global warming or climate change as well, often as a proxy for environmental quality, finding that emissions are indeed associated with higher spending on healthcare [15,16,17,18].

### 1.1. The Environmental Quality Index

The United States Environmental Protection Agency (EPA) has developed an Environmental Quality Index (EQI) to aid in research and policymaking regarding the environment. The EQI is a county-level composite of five domain indices: air, water, land, built, and social environment. Each domain consists, in turn, of individual measures of environmental quality. Forty-three air pollutants comprise the air index. The water quality domain is constructed from fifty-two measures, including forty-six water pollutants and six measures of water quantity and access. The land domain includes eighteen measures, such as the number of pesticides applied, farming acreage, agricultural animal density, and radon designation. The built environment index, consisting of sixteen individual measures, includes data on the diversity of businesses (financial, healthcare, recreational, etc.), open land and natural land cover, and walkability in addition to commuting and other transportation factors. The final element of the EQI, the social index, incorporates sixteen social variables such as median household income, median household value, the unemployment rate, violent crime rate, education, and income inequality. To construct the EQI, the EPA used a two-step process. First, individual measures were evaluated and combined via PCA (Principal Component Analysis) to compute the index for each domain, and then the domain indices were combined in a second PCA process to calculate the overall EQI. The final product is an index that ranges from −3 (good environmental quality) to 3 (poor environmental quality).

There is precedent for the use of the EQI in health-related research, as use in health research is one of the stated goals for the EQI by the EPA. The EQI has been shown to affect infant mortality rates [19], rates of multiple types of adult mortality [3], asthma prevalence [20], and diabetes incidence [21]. Air temperature is also a crucial indicator of environmental quality (and one that is missing from the EQI), as excessive cold or heat can cause higher morbidity and therefore likely drive higher healthcare utilization and spending, especially in the elderly [22].

### 1.2. The Current Study

To date, no studies have analyzed how total environmental quality affects healthcare spending, nor have any known studies examined air temperatures as a continuous variable and healthcare spending. Moreover, environmental quality and temperature have not been considered simultaneously regarding healthcare spending. These factors could be important determinants of spending levels in addition to the commonly accepted determinants such as health status [23], race [24], and income, education, and healthcare provider density [25]. This study aims to fill this gap in the literature with a spatial analytical approach on county-level data, focusing on an Environmental Quality Index and summer season heatwave days, while controlling for sociodemographic and healthcare access variables. Additionally, this study investigates the relationship between individual domain components of the overall environmental index, which includes air, water, land, social, and built environment indices. Knowing how the environment extends from affecting health to affecting healthcare expenditure levels in an ecological study with county data could point researchers and officials toward a fuller understanding of what exactly determines how money is spent on healthcare.

## 2. Methods

### 2.1. Study Design

This study used an ecological study design with the US county as the unit of analysis. Counties are administrative and political entities smaller than states but larger than cities. The US consists of 3141 counties averaging 105,000 residents and 1200 square miles in area [26]. The study area was 3068 counties of the lower forty-eight states of the US for which data are available for all variables.

### 2.2. Data

Healthcare spending: The dependent variable in this study was per capita Medicare spending for the years 2007 through 2010, geographically standardized and risk-adjusted [27]. Medicare is a public health insurance program administered by the United States Department of Health and Human Services that provides coverage to Americans over the age of 65 and some qualifying disabled persons. Geographic standardization adjusts the dollar value of the expenditures by local economic characteristics to account for the large differences in purchasing power between, for example, San Francisco, California, and rural Mississippi. Adjusting the spending by health risk controls for the differences in spending between different health risk levels of county populations. Medicare spending data are the only data available at the county level nationally.

Environmental quality: To represent environmental quality, I selected the 2006–2010 version of the Environmental Quality Index (EQI), described above in the Introduction, developed by the US Environmental Protection Agency. The EPA’s EQI data can be found online at https://www.epa.gov/healthresearch/environmental-quality-index-eqi#downloads (Accessed on 1 September 2020).

Temperature: For the second independent variable of interest, two measures of extreme heat events were chosen to test the robustness of the results to different measures of heat events. The first measure selected was the average number of heatwave days in May through September during the years of 2006 to 2010 (denoted as HWD in the Results section). A heatwave day is defined as a day above the 95th percentile of the maximum daily heat index for that county. The second measure included was the air temperature adjusted for ambient humidity, or heat index (HeatIndex). The data source for these variables comes from the National Climate Assessment portal of the CDC WONDER database [28].

### 2.3. Covariates

Medicare beneficiary characteristics: Beneficiary demographic and health characteristics have been found to be important factors that influence Medicare spending levels [29]. The median age (abbreviated as Age), percent female (Female), percent White/Caucasian (White), percent eligible for Medicaid (Medicaid), and Health Condition Category (HCC) of Medicare beneficiaries were included in analyses to minimize potential confounding. Data for these variables were provided by the CMS Geographic Public Use File (same as healthcare spending data source). Additionally, median household income for all residents was included as a control variable, and the data for this variable were taken from the US Census Bureau [30].

Healthcare access: The availability and access to medical services and products determines to a large degree how much is spent on healthcare. This is especially true of Medicare spending [31]. To control for healthcare access, I include the number of doctors (abbreviated to Doctors), hospitals (Hospitals), and hospital beds (Beds) per 10,000 county residents as per the Area Health Resource Files (AHRF) published by the US Department of Health and Human Services [32].

### 2.4. Statistical Analysis

I first calculated the descriptive statistics and bivariate correlations between variables. Then, scatterplot graphs with loess smoothing curves were produced to observe the linearity of the relationship between the dependent and independent variables. Next, I ran ordinary least squares models with all variables included and computed variance inflation factor (VIF) scores for each variable to assess multicollinearity [33]. Using an accepted VIF score threshold of 5.0 for the purpose of minimizing multicollinearity, I removed variables that exceeded a score of 5.0, which was only the number of hospital beds per 10,000 residents. Spatial autocorrelation was also tested for using the Moran’s I test statistic [34] with a queen contiguity pattern, due to the highly congruent distribution of counties. The OLS model did not control for spatial autocorrelation.

The final analytical stage was the execution of a general additive model (GAM) in two formulations; the first GAM iteration included just the overall EQI, while the second iteration was identical to the first except for the individual constituents (air, water, land, built, and social indices) added in place of the EQI to observe how each individual component predicts healthcare expenditures. General additive models are built on flexible mathematical functions rather than exclusively linear relationships to describe how variables relate to each other and were employed in this study for that very reason; the scatterplots of the IVs and DV show considerable variability in the trend lines (Supplemental Figures S1 and S2). The OLS and GAMs were also compared by Akaike Information Criterion (AIC) [35] to determine whether nonlinearity was a decisive factor in the modeling process. To control for spatial dependence, a smoothing term was incorporated into the model with a Markov Random Field element that adjusts for adjacency between neighboring units using 400 knots (the minimum number that yields a non-significant Moran’s I test).

Smoothing terms with 10 knots and thin plate regression splines were applied to terms with nonlinear graphic relationships with healthcare spending levels; both EQI and temperature had smoothers applied in the first iteration analysis due to apparent nonlinearity. In the second iteration, smoothers were applied to all the constituents of the EQI as well as heatwave days to account for any nonlinearity that may exist in the relationship between those variables and spending. GAMs were run in the mgcv package V1.8-33 [36] and the Moran’s I test statistic was computed in the spdep package [37].

## 3. Results

### 3.1. Exploratory Analysis

**Figure 1:**
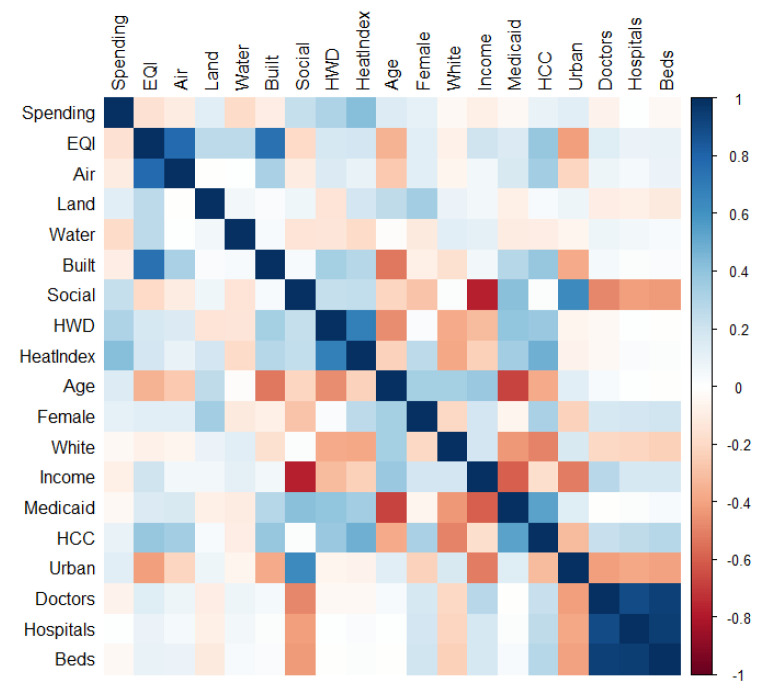
Bivariate correlation matrix for all the study variables.

Per capita Medicare spending ranged from a low of USD 5785.30 to a high of USD 14,826.40, with a mean of USD 9092.40 and a standard deviation of USD 1045.80. The EQI data, because of being constructed with PCA, follow a normal whole-unit distribution ranging from −3 to 3 with a mean of 0. Average annual air surface temperatures range from a low of 39 °F to 87 °F and a mean temperature of 63 °F. Full descriptive statistics for the entire dataset can be found in Supplemental Table S1. The Pearson bivariate correlation between EQI and per capita spending was −0.15 (*p* > 0.05), and that between temperature and spending was 0.39 (*p* > 0.05). The magnitude of all the bivariate correlations can be seen in Figure 1, which illustrates the small correlations between healthcare spending and many of the study variables, including EQI.

**Figure 2:**
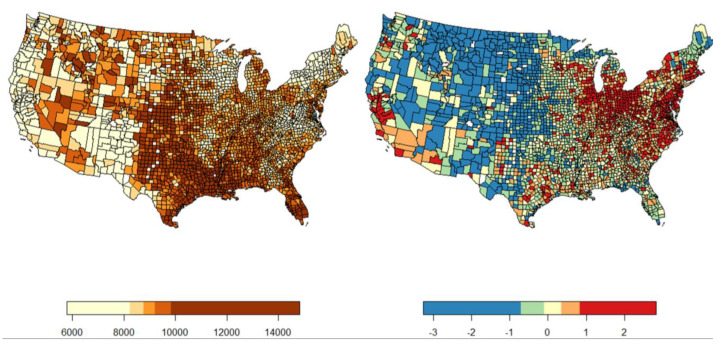
County map of per capita Medicare spending by quintile on the (**left**) and EQI on the (**right**). The central and southern portions of the country host many of the highest spending counties, with the lower-spending areas situated along the coasts, the upper Midwest, and the mountain West regions. The counties with the best EQI scores are found predominantly in the western half of the country, and the counties with the worst EQI scores are concentrated mostly in the Midwest and on the east coast of the US.

**Figure 3:**
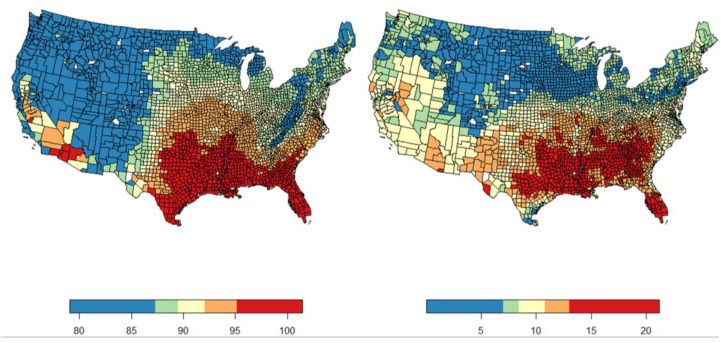
Maps of heat indexes (in degrees Fahrenheit) on the (**left**) and heatwave days on the (**right**), demarcated by county by quintile. The concentration of both measures of high temperatures has a clear geographic trend in the Southeastern United States.

The spatial distribution of the dependent and independent variables exhibits marked patterns. Per capita Medicare spending is highest in the central and southern regions of the US, most notably Kansas, Oklahoma, Texas, and Louisiana, as can be seen in Figure 2 (left). The lowest-spending counties are found on the West Coast, Northeast, and Mid-Atlantic. Regarding EQI, the counties with the lowest EQI values (indicating the best environmental quality) are in the Great Plains (Nebraska, Kansas, and the Dakotas) and the Mountain West (Colorado, Montana, Wyoming, Utah, and Idaho). The counties with the worst environmental quality are those in the Midwest (Illinois, Indiana, and Ohio specifically) and on the East Coast, including Maryland, North Carolina, and Virginia (Figure 2, right). Air temperatures follow a clear climatological delineation, with the coolest counties in the northern US and the warmest in the south (Figure 3, left and right).

The scatterplots with loess curves demonstrate the nonlinearity of the relationships between EQI and Medicare expenditures and air temperatures and expenditures. The nonlinear nature of the bivariate relationship between EQI and spending (Supplemental Figure S1) is slight but evident, as the lowest and highest values of the EQI range are related to the lowest spending levels. The association between both the measure of high temperatures and spending is clearly nonlinear (Supplemental Figure S2), with distinct segments of the graph showing highly divergent slopes.

### 3.2. Main Results

**Figure 4:**
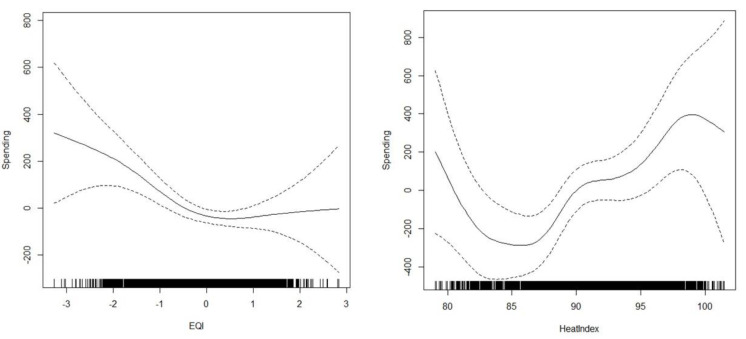
Graph of the nonlinear smoothing term on EQI and spending (**left**) and HeatIndex and spending (**right**). The multifaceted and regimented association between EQI and spending is clear in the left graph, denoting that lower spending levels are seen in the middle of the EQI range.

The GAM with overall environmental quality (just the EQI variable) and temperature identified those variables as significantly associated with county healthcare spending (Table 1). EQI, with a smoothing term, was statistically significant (*p* = 0.004), as was the smoothed HeatIndex (*p* = 0.001). Other variables in the model that were statistically significant at the commonly accepted levels were Doctors, Hospitals, Age, Female, and Medicaid. The nonlinearity of the smoothing terms on EQI (mild nonlinearity) and temperature (pronounced nonlinearity) can be seen in Figure 4. The variability of the association is evident for EQI and especially for temperature. The graph of the EQI results shows a bifurcated trend in higher spending at the extremes and lower spending toward the central zero value. Heatwave days were not statistically significant (*p* = 0.354). The first GAM also controlled for spatial autocorrelation per the Moran’s I statistic test (coefficient = 0.01, *p* value = 0.5) and produced an adjusted R-square of 0.65.

In the second GAM iteration featuring the five individual domain components of the EQI, only the social and built environment indices were statistically significant (smoothed social *p* value = 0.000, built index *p* value = 0.002), suggesting that the statistical significance of the overall EQI variable in the first series of models is driven in part or entirely by the social and built components. The full results of the second GAM can be seen in Table 2. The second model also controlled for spatial autocorrelation (Moran’s I statistic = −0.01, *p* value = 0.7), meaning that the values of the variables in each county were not unduly influenced by the values of the variables in neighboring counties, and had an adjusted R-square of 0.66.

## 4. Discussion

These results show a statistically significant, nonlinear, and varying association between the Environmental Quality Index and healthcare spending at the county level, a significant association between the heat index and spending, and an association between the number of heatwave days and spending that is not statistically significant. These results are mostly consistent with previous studies that have found that environmental degradation (Davies, 2006) has the potential to drive healthcare costs and spending up. The finding that average maximum heat index is significantly associated with spending aligns with extant research on spending and extreme air temperatures [14].

The individual constituent domains of the EQI held unexpected associations with healthcare spending, indicating that the integrated status of the EQI is perhaps not optimally useful in capturing overall environmental quality and that other determinants of healthcare spending exert more influence on spending than the environment does. The air, water, and land indices were not statistically significant predictors of spending, regardless of if a nonlinear smoother was applied or not. The social and built environment indices did, however, have a significant association with spending. This could be explained by the crucial importance of societal factors [24,25] and both physical and service infrastructure in determining healthcare costs and spending [38,39]. For example, the social domain of the Environmental Quality Index contains both income (in the form of median household income) and education (percent of county residents with at least a bachelor’s degree), both of which Thornton and Rice [25] found to be significant predictors of healthcare expenditures. It is also possible, even probable, that high air temperatures may be driving higher healthcare spending in places where there are also the high EQI scores the denote poor environmental quality.

It is unanticipated that air, water, and land domain indices were not significantly associated with healthcare spending. These results do not align with much of the previous work on specific domains or contaminants (e.g., [5,6,11,12]). The non-significance of the air, water, and land associations could result from the measure used; the quality indices for each of these facets are not associated with spending, but individual components of the domains, such as by the nitrogen oxide (NO and NO_2_) air pollutants, groundwater bacterial levels, or soil radon levels, might be. Although it is beyond the scope of this study, further research that addresses these limitations or utilizes more highly resolved or individual environmental factors is called for to explore these unanticipated results.

These findings contribute to the existing literature by identifying the environment, as measured by both an index of environmental quality and air temperatures, as an important determinant of per capita county healthcare expenditures. These results can be used to inform further research with more resolved data or, ideally, individual-level data or different measures of environmental factors. The results of this study might also be of interest to healthcare experts, health policymakers, and health insurers—especially the Centers for Medicare and Medicaid Services, who should be cognizant of the role the environment plays in shaping the financial landscape of healthcare in America.

### Strengths and Limitations

The chief strength of this study lies in the independent variables of interest and the total environmental quality in the form of a comprehensive index, the latter including dangerous air temperatures. This study also encompasses almost the entire contiguous United States (99% of all counties). Due to the wealth of data available at the county level, important confounders like health service, health status, and socioeconomic factors were able to be controlled for. The analytical approach also had the strength of using a model that simultaneously accounted for nonlinearity and spatial autocorrelation, both of which can drastically affect the interpretability of statistical models [40,41].

Several limitations also exist for this study. First and foremost is the ecological fallacy, meaning that conclusions drawn from aggregated data cannot be applied to individuals. This study can, and does, only generalize its findings to counties or larger political units. Additionally, the measure of healthcare spending in this study was per capita Medicare spending. Medicare covers the elderly (those age sixty-five and over) and those with that have qualifying disabilities, so only a portion of the total American populace is considered. Generalizing these findings to the public or to non-Americans should therefore be carried out with caution.

## 5. Conclusions

The results of this study show a nonlinear and varying association between the Environmental Quality Index and healthcare spending and a generally positive association between increasing air temperatures and healthcare spending at the county level in a spatial general additive model. Two individual domains that constitute the EQI, social and built environment indices, were also found to be significant in a subsequent model. These results likely stand as the first examination of how total environmental quality relates to healthcare spending. Governments, policymakers, and public health professionals, considering these findings, might focus on bolstering the social and built capital of areas as a way of reducing healthcare costs. To improve upon this research and perhaps make conclusions about individual persons rather than large units like counties, future research should be undertaken with more highly resolved data and other measures of environmental quality.

## Figures and Tables

**Table 1:** Model results from the GAM first iteration with EQI, temperature, and covariates.

	Heatwave Days		HeatIndex	
	Estimate	*p* Value	Estimate	*p* Value
Smoothed				
EQI	NA	0.004	NA	0.006
Heat	NA	0.354	NA	0.001
Geosmoother	NA	0.000	NA	0.000
Linear				
Age	115.21	0.000	116.1	0.000
Female	−32.84	0.000	−32.1	0.000
White	6.21	0.000	6.9	0.000
Medicaid	−1.40	0.691	−1.6	0.651
HCC	895.63	0.002	837.7	0.005
Income	0.01	0.010	0.0	0.031
Doctors	−0.46	0.001	−0.4	0.003
Hospitals	34.81	0.000	31.5	0.000
Urban	59.48	0.000	59.5	0.000
Fit statistics				
R-sq.(adj)	0.65		0.65	
Deviance explained	0.68		0.69	
Moran’s I	0.00	0.5	0.00	0.4

**Table 2:** Results of the second iteration GAM with the individual constituents (air, water, land, build, and social environments) of the EQI, air temperature, and covariates.

	Estimate	*p* Value
Smoothed		
Air	NA	0.930
Water	NA	0.920
Land	NA	1.000
Built	NA	0.002
Social	NA	0.000
Heat	NA	0.320
Geosmoother	NA	0.000
Linear		
Age	78.40	0.000
Female	1.05	0.909
White	5.24	0.001
Medicaid	−12.54	0.001
HCC	1081.61	0.000
Income	0.01	0.000
Doctors	−0.63	0.000
Hospitals	48.41	0.000
Urban	19.34	0.159
Fit statistics		
R-sq.(adj)	0.66	
Deviance explained	0.70	
Moran’s I	−0.01	0.7

## Data Availability

The data used in this study are available upon request from the author.

## Supplementary Materials

The following supporting information can be downloaded at https://www.mdpi.com/article/10.3390/ijerph21101322/s1: Figure S1: Scatterplot of EQI against healthcare spending with smoothed loess curves with 10 inflection points; Figure S2: Scatterplots of heat index (left) and heat wave days (right) compared against spending, with a loess smoothing curve; Table S1: Descriptive statistics for the study dataset, excluding the ranked variable for urbanization.

## References

[1] Brender J.D., Maantay J.A., Chakraborty J. (2011). Residential proximity to environmental hazards and adverse health outcomes. Am. J. Public Health.

[2] Kim K.H., Kabir E., Kabir S. (2015). A review on the human health impact of airborne particulate matter. Environ. Int..

[3] Jian Y., Messer L.C., Jagai J.S., Rappazzo K.M., Gray C.L., Grabich S.C., Lobdell D.T. (2017). Associations between environmental quality and mortality in the contiguous United States, 2000–2005. Environ. Health Perspect..

[4] Remoundou K., Koundouri P. (2009). Environmental effects on public health: An economic perspective. Int. J. Environ. Res. Public Health.

[5] Sætterstrøm B., Kruse M., Brønnum-Hansen H., Bønløkke J.H., Meulengracht Flachs E., Sørensen J. (2012). A method to assess the potential effects of air pollution mitigation on healthcare costs. J. Environ. Public Health.

[6] Roy A., Sheffield P., Wong K., Trasande L. (2011). The effects of outdoor air pollutants on the costs of pediatric asthma hospitalizations in the United States, 1999–2007. Med. Care.

[7] Lightwood J., Glantz S.A. (2016). Smoking behavior and healthcare expenditure in the United States, 1992–2009: Panel data estimates. PLoS Med..

[8] Yao T., Sung H.Y., Wang Y., Lightwood J., Max W. (2018). Healthcare costs attributable to secondhand smoke exposure at home for US adults. Prev. Med..

[9] Huovinen E., Laine J., Virtanen M.J., Snellman M., Hujanen T., Kiiskinen U., Kujansuu E., Lumio J., Ruutu P., Kuusi M. (2013). Excess healthcare costs of a large waterborne outbreak in Finland. Scand. J. Public Health.

[10] Corso P.S., Kramer M.H., Blair K.A., Addiss D.G., Davis J.P., Haddix A.C. (2003). Costs of illness in the 1993 waterborne Cryptosporidium outbreak, Milwaukee, Wisconsin. Emerg. Infect. Dis..

[11] DeFlorio-Barker S., Wing C., Jones R.M., Dorevitch S. (2018). Estimate of incidence and cost of recreational waterborne illness on United States surface waters. Environ. Health.

[12] Alzahrani F., Collins A.R., Erfanian E. (2020). Drinking water quality impacts on health care expenditures in the United States. Water Resour. Econ..

[13] Noe R.S., Jin J.O., Wolkin A.F. (2012). Exposure to natural cold and heat: Hypothermia and hyperthermia Medicare claims, United States, 2004–2005. Am. J. Public Health.

[14] Wondmagegn B.Y., Xiang J., Williams S., Pisaniello D., Bi P. (2019). What do we know about the healthcare costs of extreme heat exposure? A comprehensive literature review. Sci. Total Environ..

[15] Abdullah H., Azam M., Zakariya S.K. (2016). The impact of environmental quality on public health expenditure in Malaysia. Asia Pac. J. Adv. Bus. Soc. Stud..

[16] Alimi O.Y., Ajide K.B., Isola W.A. (2019). Environmental quality and health expenditure in ECOWAS. Environ. Dev. Sustain..

[17] Ibukun C.O., Osinubi T.T. (2020). Environmental Quality, Economic Growth, and Health Expenditure: Empirical Evidence from a Panel of African Countries. Afr. J. Econ. Rev..

[18] Marsh K., Ganz M., Nørtoft E., Lund N., Graff-Zivin J. (2016). Incorporating environmental outcomes into a health economic model. Int. J. Technol. Assess. Health Care.

[19] Patel A.P., Jagai J.S., Messer L.C., Gray C.L., Rappazzo K.M., Deflorio-Barker S.A., Lobdell D.T. (2018). Associations between environmental quality and infant mortality in the United States, 2000–2005. Arch. Public Health.

[20] Gray C.L., Lobdell D.T., Rappazzo K.M., Jian Y., Jagai J.S., Messer L.C., Patel A.P., De Florio-Barker S.A., Lyttle C., Solway J. (2018). Associations between environmental quality and adult asthma prevalence in medical claims data. Environ. Res..

[21] Jagai J.S., Krajewski A.K., Shaikh S., Lobdell D.T., Sargis R.M. (2020). Association between environmental quality and diabetes in the USA. J. Diabetes Investig..

[22] Bunker A., Wildenhain J., Vandenbergh A., Henschke N., Rocklöv J., Hajat S., Sauerborn R. (2016). Effects of air temperature on climate-sensitive mortality and morbidity outcomes in the elderly; a systematic review and meta-analysis of epidemiological evidence. eBioMedicine.

[23] Hadley J., Waidmann T. (2006). Health insurance and health at age 65: Implications for medical care spending on new Medicare beneficiaries. Health Serv. Res..

[24] Charron-Chénier R., Mueller C.W. (2018). Racial disparities in medical spending: Healthcare expenditures for black and white households (2013–2015). Race Soc. Probl..

[25] Thornton J.A., Rice J.L. (2008). Determinants of healthcare spending: A state level analysis. Appl. Econ..

[26] United State Census Bureau (2019). County Population Totals; 2010–2019. https://www.census.gov/data/tables/time-series/demo/popest/2010s-counties-total.html.

[27] Centers for Medicare Medicaid Services (2019). Rural–Urban Disparities in Health Care in Medicare.

[28] National Climate Assessment (2022). Extreme Heat Events: Heat Wave Days in May—September for years 1981–2010 on CDC WONDER Online Database, Released 2015. http://wonder.cdc.gov/NCA-heatwavedays-historic.html.

[29] Zuckerman S., Waidmann T., Berenson R., Hadley J. (2010). Clarifying sources of geographic differences in Medicare spending. N. Engl. J. Med..

[30] United States Census Bureau (2022). US Census. https://www.census.gov/en.html.

[31] Kelley A.S., Ettner S.L., Morrison R.S., Du Q., Wenger N.S., Sarkisian C.A. (2011). Determinants of medical expenditures in the last 6 months of life. Ann. Intern. Med..

[32] Health Resources and Services Administration (2018). Area Health Resource Files. https://datawarehouse.hrsa.gov/topics/ahrf.aspx.

[33] Mansfield E.R., Helms B.P. (1982). Detecting multicollinearity. Am. Stat..

[34] Moran P.A. (1950). A test for the serial independence of residuals. Biometrika.

[35] Akaike H. (1974). A new look at the statistical model identification. IEEE Trans. Autom. Control.

[36] Wood S.N. (2011). Fast stable restricted maximum likelihood and marginal likelihood estimation of semiparametric generalized linear models. J. R. Stat. Soc. B.

[37] Bivand R., Wong D.W.S. (2018). Comparing implementations of global and local indicators of spatial association. TEST.

[38] Trubka R., Newman P., Bilsborough D. (2010). The costs of urban sprawl–Infrastructure and transportation. Environ. Des. Guide.

[39] Yancey A.K., Fielding J.E., Flores G.R., Sallis J.F., McCarthy W.J., Breslow L. (2007). Creating a robust public health infrastructure for physical activity promotion. Am. J. Prev. Med..

[40] Garrido M.M., Deb P., Burgess J.F., Penrod J.D. (2012). Choosing models for health care cost analyses: Issues of nonlinearity and endogeneity. Health Serv. Res..

[41] Hawkins B.A., Diniz-Filho J.A.F., Mauricio Bini L., De Marco P., Blackburn T.M. (2007). Red herrings revisited: Spatial autocorrelaton and parameter estimation in geographical ecology. Ecography.

