# Supplementary material for: Environmental Quality, Extreme Heat, and Healthcare Expenditures
Source: Int J Environ Res Public Health. 2024 Oct 5;21(10):1322. doi: 10.3390/ijerph21101322 (PMC11507527; doi:10.3390/ijerph21101322)
Supplement: Supplementary file 1 [file ijerph-21-01322-s001.zip › ijerph-3205537-supplementary.pdf]

Supplemental Materials

Table S1. Descriptive statistics for the study dataset, excluding the ranked variable for urbanization.

Variable	Min.	Max.	Median	Mean	SD
Spending	5785.3	14826.4	8990.4	9092.4	1045.8
EQI	-3.3	2.8	0.1	0.0	0.9
Air	-2.8	3.7	0.1	0.0	0.9
Land	-3.9	1.8	0.2	0.1	0.9
Water	-1.5	2.0	0.3	0.0	1.0
Built	-3.2	3.8	0.1	0.0	1.0
Social	-5.1	2.8	0.2	0.0	1.0
HWD	0.0	21.2	9.4	9.9	3.4
HeatIndex	79.1	101.5	90.6	90.9	4.4
Age	63.0	77.0	71.5	71.5	2.0
Female	42.3	62.0	55.3	54.9	2.5
White	9.6	99.2	89.2	83.3	16.3
Income	20,990	119,525	41,864	43,715	11,022
Medicaid	3.0	71.0	19.8	21.5	8.9
HCC	0.7	1.4	0.9	0.9	0.1
Urban	1.0	6.0	5.0	4.6	1.5
Doctors	0.0	6947.0	12.0	72.0	248.8
Hospitals	0.0	111.0	1.0	2.0	4.1
Beds	0.0	25763.0	59.0	309.3	1000.9

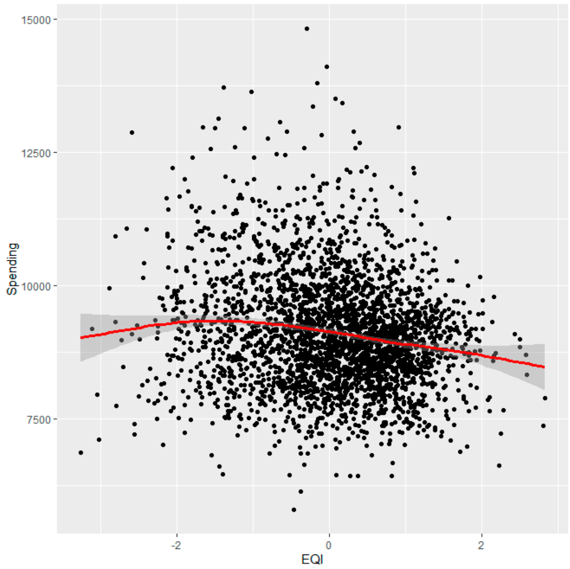

Figure S1. Scatterplot of EQI against healthcare spending with smoothed loess curves with 10 inflection points.

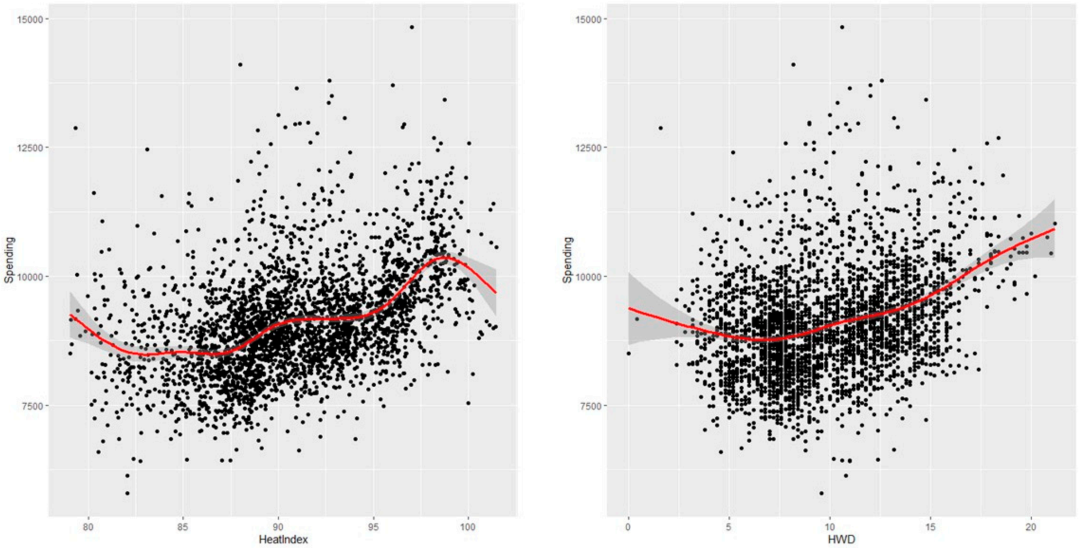

Figure S2. Scatterplots of heat index (left) and heat wave days (right) compared against spending, with a loess smoothing curve.
